# Endothelial APC/PAR1 distinctly regulates cytokine-induced pro-inflammatory VCAM-1 expression

**DOI:** 10.3389/fmolb.2023.1211597

**Published:** 2023-08-24

**Authors:** Cierra A. Birch, Helen Wedegaertner, Lennis B. Orduña-Castillo, Monica L. Gonzalez Ramirez, Huaping Qin, JoAnn Trejo

**Affiliations:** ^1^ Department of Pharmacology, School of Medicine, University of California, San Diego, CA, United States; ^2^ Biomedical Sciences Graduate Program, University of California, San Diego, CA, United States

**Keywords:** GPCR, cytoprotection, GRK, TNF-α, thrombin

## Abstract

**Introduction:** Dysfunction of the endothelium impairs its’ protective role and promotes inflammation and progression of vascular diseases. Activated Protein C (APC) elicits endothelial cytoprotective responses including barrier stabilization, anti-inflammatory and anti-apoptotic responses through the activation of the G protein-coupled receptor (GPCR) protease-activated receptor-1 (PAR1) and is a promising therapeutic. Despite recent advancements in developing new Activated protein C variants with clinical potential, the mechanism by which APC/PAR1 promotes different cytoprotective responses remains unclear and is important to understand to advance Activated protein C and new targets as future therapeutics. Here we examined the mechanisms by which APC/PAR1 attenuates cytokine-induced pro-inflammatory vascular cell adhesion molecule (VCAM-1) expression, a key mediator of endothelial inflammatory responses.

**Methods:** Quantitative multiplexed mass spectrometry analysis of Activated protein C treated endothelial cells, endothelial cell transcriptomics database (EndoDB) online repository queries, biochemical measurements of protein expression, quantitative real-time polymerase chain reaction (RT-qPCR) measurement of mRNA transcript abundance, pharmacological inhibitors and siRNA transfections of human cultured endothelial cells.

**Results:** Here we report that Activated Protein C modulates phosphorylation of tumor necrosis factor (TNF)-α signaling pathway components and attenuates of TNF-α induced VCAM-1 expression independent of mRNA stability. Unexpectedly, we found a critical role for the G protein-coupled receptor co-receptor sphingosine-1 phosphate receptor-1 (S1PR1) and the G protein receptor kinase-2 (GRK2) in mediating APC/PAR1 anti-inflammatory responses in endothelial cells.

**Discussion:** This study provides new knowledge of the mechanisms by which different APC/PAR1 cytoprotective responses are mediated through discrete β-arrestin-2-driven signaling pathways modulated by specific G protein-coupled receptor co-receptors and GRKs.

## 1 Introduction

Endothelial dysfunction, a hallmark of inflammation, is linked to the pathogenesis of vascular diseases and results in barrier disruption, inflammation and apoptosis (Pober and Sessa, 2007). There are currently limited treatment options for improving endothelial dysfunction resulting in high morbidity and mortality (Dombrovskiy et al., 2007; Goldenberg et al., 2011). Activated Protein C (APC), a natural anti-coagulant protease, is a promising therapeutic and exhibits multiple pharmacological benefits in preclinical studies of injury and inflammation (Kerschen et al., 2007; Griffin et al., 2015; Griffin et al., 2018). In endothelial cells, APC elicits several cytoprotective responses including barrier stabilization, anti-inflammatory and anti-apoptotic activities (Riewald and Ruf, 2005; Mosnier et al., 2007; Molinar-Inglis et al., 2021). Protease-activated receptor-1 (PAR1), a G protein-coupled receptor (GPCR), is the central mediator of APC cellular signaling in endothelial cells.

Activation of PAR1 occurs through irreversible proteolytic cleavage of its’ N-terminus, generating a new N-terminal tethered ligand sequence that binds intramolecularly to the receptor to trigger transmembrane signaling. The classic mechanism of PAR1 activation established for the coagulant protease thrombin occurs through cleavage at the arginine (R)-41 site within the N-terminus (Vu et al., 1991). However, APC bound to endothelial protein C receptor activates PAR1 via cleavage at an N-terminal R-46 site, generating a distinct tethered ligand that drives endothelial cytoprotection (Mosnier et al., 2012; Sinha et al., 2018). In addition, APC/PAR1 localization to caveolae, caveolin-1 enriched microdomains, abundant in endothelial cells is required for cytoprotective signaling (Bae et al., 2008; Russo et al., 2009; Birch et al., 2021; Molinar-Inglis et al., 2021). We discovered that APC/PAR1 signals primarily via β-arrestin-2 rather than heterotrimeric G proteins to promote endothelial barrier stabilization (Soh and Trejo, 2011) and protection against cytokine-induced apoptosis (Molinar-Inglis et al., 2021). β-arrestin-2 has also been implicated in mediating APC/PAR1 endothelial anti-inflammatory responses *in vitro* and *in vivo* (Roy et al., 2016; Kanki et al., 2019). However, there remains a limited understanding of the diverse mechanisms by which APC-activation of PAR1 promotes endothelial cytoprotection.

β-arrestin interaction with activated GPCRs is mediated by receptor phosphorylation and engagement with the transmembrane core (Shenoy and Lefkowitz, 2011; Shukla et al., 2011). G protein receptor kinases (GRKs) phosphorylate activated GPCRs and differentially modulate β-arrestin biased signaling (Kim et al., 2005; Ren et al., 2005; Drube et al., 2022). Interaction with other GPCRs can also alter active GPCR conformations, phosphorylation, β-arrestin engagement and biased signaling (Shen et al., 2018). Indeed, co-receptor interaction controls diverse APC/PAR1-induced β-arrestin driven endothelial cytoprotective responses. APC/PAR1 requires the co-receptor PAR3 for endothelial barrier protection (Burnier and Mosnier, 2013), whereas we made the unexpected discovery that the sphingosine-1 phosphate receptor-1 (S1PR1) is required for APC/PAR1 anti-apoptotic activities (Molinar-Inglis et al., 2021). These studies suggest that discrete GPCR complexes may uniquely function in APC-stimulated recruitment of GRKs to promote distinct β-arrestin-2 driven cytoprotective responses. This also raises the question of whether different co-receptors function in APC/PAR1-induced anti-inflammatory responses, which is not known.

In the present study, we examined the mechanisms by which APC modulates tumor necrosis factor-α (TNF-α) cytokine induced inflammatory responses in human cultured endothelial cells. We show that APC stimulation alters phosphorylation of several TNF-α signal pathway components and attenuates of TNF-α induced vascular cell adhesion molecule-1 (VCAM-1) expression independent of mRNA stability. Unexpectedly, we found a role for S1PR1 and GRK2 in APC/PAR1 mediated suppression of cytokine-induced VCAM-1 expression. This study reveals that different APC/PAR1 cytoprotective responses are mediated by discrete β-arrestin-2-driven signaling pathways modulated by specific co-receptors and GRKs.

## 2 Materials and methods

### 2.1 Cell culture and treatments

HUVEC-derived endothelial EA. hy926 cells (ATCC, #CRL-2922) were grown at 37°C in 8% CO_2_ in Dulbecco’s Modified Eagle Medium (DMEM) (Gibco, #10–013-CV and #10437–028) supplemented with 10% fetal bovine serum (FBS) and 20% preconditioned media as we previously described (Molinar-Inglis et al., 2021). Endothelial cell confluent monolayers were washed with phosphate buffered saline (PBS) and grown in low-serum 0.4% FBS DMEM overnight. Cells were then washed and serum starved in DMEM containing 10 mM HEPES, 1 mM CaCl_2_, and 1 mg/mL bovine serum albumin (BSA) for 1 h prior to agonist, antagonist and/or inhibitor treatments at 37°C.

Endothelial EA. hy926 cells were pretreated with or without 20 nM APC for 3 h at 37°C (Prolytix, #HCAPC-0080). Cells were then stimulated with 10 ng/mL TNF-α (PeproTech, #300–01A) for the indicated times at 37°C and responses measured. To block PAR1 signaling, serum-starved cells were pretreated at 37°C with 10 µM Vorapaxar (Axon Medchem, #1755) for 1 h before APC treatment. S1PR1 signaling was inhibited by incubation with 10 µM W146 (Tocris, #3602) for 30 min at 37°C. GRK2 was inhibited by preincubating endothelial cells with 50 µM Compound (CPMD)101 (Sigma-Aldrich, #SML2784) at 37°C for 1 h in serum-free media before APC stimulation.

### 2.2 Antibodies

The following antibodies were used in this study: anti-VCAM-1 (#13662S), anti-ICAM-1 (#4915S)**,** anti-phospho-p38 T180/Y182 (#4511) and anti-p38 MAPK (#8690) antibodies (Cell Signaling Technology), anti-β-actin (Sigma-Aldrich, #5316), anti-GRK2/3 (Millipore, #05465), anti-GRK4-6 (Millipore, #05466) and anti-GAPDH (GeneTex, #GT239). Secondary antibodies were anti-mouse or anti-rabbit HRP conjugated antibodies (Bio-Rad, #170–6516 and #170–6515).

### 2.3 Endothelial cell database (EndoDB) analysis

To explore the effect of TNF-α on adhesion molecule and GRK mRNA expression in endothelial cells, we examine the online publicly available endothelial cell database (EndoDB) (https://endotheliomics.shinyapps.io/endodb/) (Khan et al., 2019), which contains normalized and curated RNA-seq datasets collected from the Gene Expression Omnibus and ArrayExpress repositories. We examined four independent studies from HUVEC (#1, E-GEOD-8166_P2; #2, E-SGRP-3; #3, E-GEOD-2639 and #4, E-GEOD-9055) and one from aorta derived endothelial cells (#5, E-MEXP-3540). Data were analyzed for changes in mRNA transcript levels in control cells and TNF-α treated cells for the adhesion molecules *VCAM1, ICAM*1 and *SELE*, as well as for the GRKs (*GRK2, GRK3, GRK5, GRK6*). The relative mRNA levels, including replicates, of each of the 5 independent studies were analyzed by box-and-whisker plot with median and range from minimum-to-maximum.

### 2.4 VCAM-1 and ICAM-1 adhesion molecule protein expression detection

Endothelial EA. hy926 cells were seeded in 12-well plates at 2.8 × 10^5^ cells per well, grown overnight and switched to low serum (0.4% FBS) for 24 h 37°C. Cells were washed, incubated with DMEM containing 10 mM HEPES, 1 mM CaCl_2_, and 1 mg/mL BSA for 1 h and then pretreated with 20 nM APC for 3 h followed by TNF-α (10 ng/mL) stimulation for 24 h at 37 °C. Cells were lysed in RIPA buffer (25 mM Tris-HCl, pH 7.6, 150 mM NaCl, 1% NP40, 0.5% sodium deoxycholic acid, 0.1% SDS) supplemented with protease inhibitors (1 mM PMSF, 2 μg/mL Aprotinin, 10 μg/mL Leupeptin, 1 μg/mL Pepstatin, and 1 μg/mL Trypsin protease inhibitor). Equivalent amounts of cell lysates were immunoblotted with either VCAM-1, ICAM-1, GRK2,3, GRK4,5,6 or GAPDH antibodies, developed by chemiluminescence and quantified by densitometry analysis using ImageJ software.

### 2.5 mRNA stability assay

Endothelial EA. hy926 cells were seeded in 6-well plates at 3.2 × 10^5^ cells per well, cultured and serum-starved and pretreated with 20 nM APC for 3 h, followed by incubation with TNF-α (10 ng/mL) for 24 h 37°C. Cells were then washed with PBS and then left untreated (0 h) or treated with 10 μg/mL Actinomycin D for 2, 4, 5 and 6 h at 37°C. RNA was extracted and *VCAM1* mRNA transcripts quantified by RT-qPCR using a TaqMan Gene Expression Probe (*VCAM1*, #Hs01003372_m1 ThermoFisher) as described below.

### 2.6 SiRNA transfections

Cells were seeded at 1.4 × 10^5^ cells per well in a 12-well plate, grown overnight, and transfected with siRNA using the TransIT-X2 System (Mirus, #MIR 600) according to the manufacturer’s instructions. Experiments were conducted 48 h post transfection. The following siRNAs were used in these studies: 25 nM GRK2 #1 siRNA 5′-CCG​GGA​GAT​CTT​CGA​CTC​ATA-3′ (Qiagen #SI00287378), 25 nM GRK5 siRNA #5 5′-AGC​GTC​ATA​ACT​AGA​ACT​GAA-3′ (Qiagen, #SI00287770), 25 nM of PAR1-208 siRNA 5′-AGA​UUA​GUC​UCC​AUC​AAU​A-3′ (Morris et al., 2006), 25 nM PAR3 siRNA #6 5′-CAC​TTA​ATG​CAT​ACG​ATC​ATA-3′ (Qiagen, #SI03062486) and AllStars Negative Control non-specific siRNA (Qiagen, #1027281).

### 2.7 Quantitative reverse transcription polymerase chain reaction (RT-qPCR)

Endothelial EA. hy926 cells were seeded in 6-well plates at 3.2 × 10^5^ cells per well, grown, cultured and treated as described above. Cells were wash with PBS and RNA extracted using the kit Direct-zol RNA Miniprep (Zymo Research, #R2050) according to according to the manufacturer’s instructions. RNA was quantified and cDNA synthesized from 1 μg RNA using SuperScript IV VILO Master Mix with ezDNase enzyme kit (#111766050, ThermoFisher Scientific). Quantitative RT-PCR was performed with TaqMan Fast Advanced Master Mix (#4444964, ThermoFisher Scientific) and TaqMan Gene Expression Probes including: PAR1 (*F2R*) (ThermoFisher, #Hs05045041_s1), PAR3 *(F2RL3*) (ThermoFisher, #Hs00187982_m1), VCAM-1 (*VCAM1*) (ThermoFisher, #Hs01003372_m1) and TNF-α (ThermoFisher, #Hs00174128_m1). The mRNA transcript levels were normalized to 18S expression and analyzed using the comparative CT (threshold cycle) method.

### 2.8 Bioluminescence resonance energy transfer (BRET) assay

HEK293 cells were purchased from ATCC and maintained at 37°C in 5% CO_2_ in Dulbecco’s Modified Eagle Medium (DMEM) (Gibco #10–013-CV) supplemented with 10% fetal bovine serum (FBS) (Gibco #10437–028). HEK293 cells were seeded at 2.5 × 10^5^ cells per well of a 12-well plate, grown overnight, and then transfected with BRET donor RLuc-β-arr2 (100 ng) together with BRET acceptor PAR1-YFP (400 ng) and the APC co-receptor EPCR Halo (200 ng), with polyethylenimine (PEI) diluted in Opti-MEM. The next day, cells were pooled and re-seeded into poly-D-lysine coated 96-well plate at 3 × 10^4^ cells per well and grown overnight. The following day, cells were washed with PBS and starved for 1 h using a 1:1 equal mixture of DMEM no phenol red (Gibco, #31053–028) combined with PBS. After starvation, cells were preincubated with 5 µM of RLuc substrate Coelenterazine H (Biotium, #10111–1) for 5 min followed by the addition of 20 nM APC (Prolytix, HCAPC-0080) and BRET measurements were taken at 37°C over time. All BRET measurements were performed with a Berthold TriStar LB941 multimode plate reader running MicroWIN 2010 software (Berthold Biotechnologies) using two filter settings: 480 nm for Rluc and 530 nm for YFP. The BRET signal was calculated as the emission at 530 nm divided by the emission at 480 nm. The BRET signals were normalized to basal BRET ratios and expressed as the percent over basal.

### 2.9 Quantitative phospho-proteomics mass spectrometry analysis

APC-induced changes in phosphorylation of TNF-α signaling pathway components were determined from three biological replicates using tandem mass tag 10-plex quantitative phospho-proteomic analysis as we previously described (Lin et al., 2020). Briefly, endothelial EA. hy926 cells were grown, serum starved overnight, and stimulated with 20 nM APC for 0, 15 or 30 min and processed as described (Lin et al., 2020). Temporal changes in phosphorylation of TNF-α pathway components are displayed as heat maps, where each row is colored by relative abundance from minimum (blue) and maximum (red) intensity of the phospho-peptide abundance or not detected (N.D.) colored as gray. The data are also displayed as floating bar graphs of the average and minimum-to-maximum of the individual replicates (not including N.D.).

### 2.10 Model and prediction analysis

Adobe Illustrator and Photoshop were used to create figures. The cartoons were created with BioRender.com. The “TNF Pathway” was adapted from BioRender.com (2023) https://app.biorender.com/biorender-templates.

### 2.11 Statistical analysis

Data were analyzed using Prism software (version 9.4.1; GraphPad Software). Statistical analysis was determined by performing one-way analysis of variance (ANOVA) with multiple comparisons.

## 3 Results

### 3.1 Quantitative phospho-proteomic analysis of APC/PAR1 modulation of TNF-α signaling pathways

**FIGURE 1:**
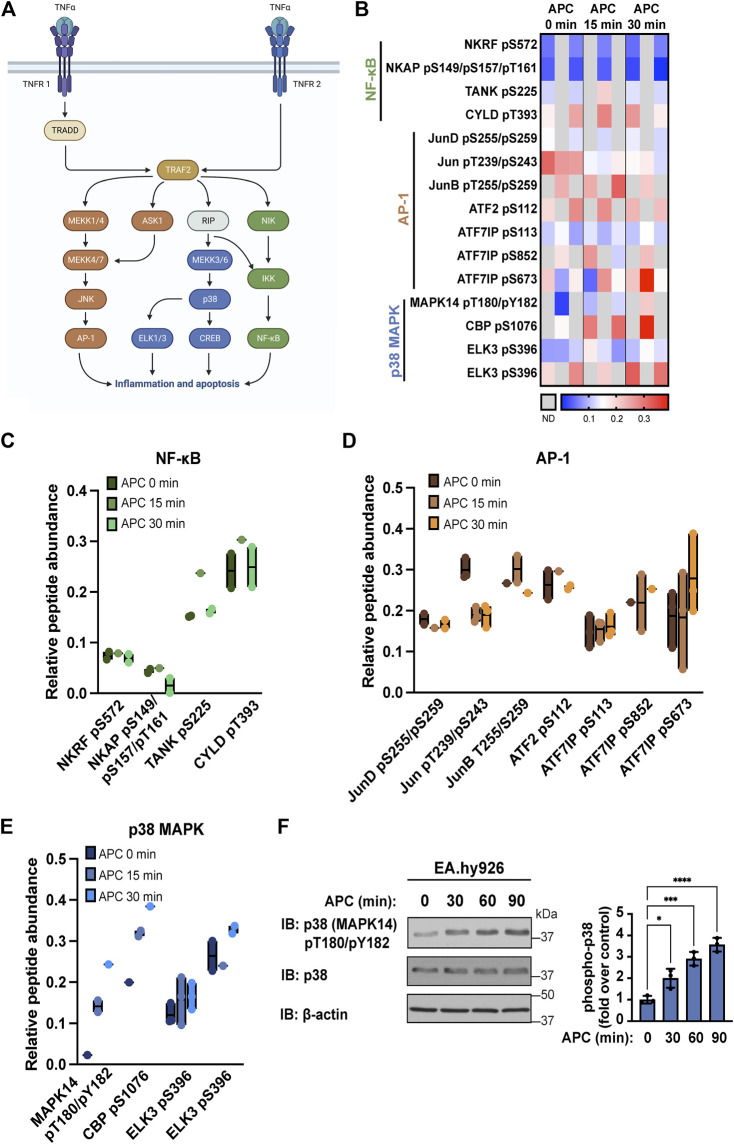
APC modulates TNF-α stimulated proinflammatory pathways in endothelial cells. **(A)** TNF-α signals through AP-1, p38 MAPK (MAPK14), and NF-κB pathways to promote inflammation and apoptosis in endothelial cells. **(B)** Heat map of TNF-α pathways components illustrates phospho-peptide abundance from endothelial cells treated with APC for the indicated times from three biological replicates, where increases and decreases in phospho-peptide abundance are indicated by the red and blue intensities, respectively and gray is not detected (N.D.). Specific changes in NF-κB **(C)**, AP-1 **(D)** and p38 MAPK **(E)** pathway components phospho-peptide abundance detected in endothelial cells treated with or without APC are shown as floating bar graphs of the average and minimum-to-maximum of the individual replicates. **(F)** APC stimulation of p38 MAPK phosphorylation in endothelial cells was validated by immunoblot. Data (mean ± S.D.) are from three independent experiments and expressed as fold over control (0 min) analyzed by ANOVA; *, *p* < 0.05; ***, *p* < 0.001; ****, *p* < 0.0001.

A key feature of APC/PAR1 cytoprotective signaling is the capacity to attenuate TNF-α induced endothelial inflammatory responses (Joyce et al., 2001; Riewald and Ruf, 2005). TNF-α, a potent cytokine, promotes multiple endothelial inflammatory responses including upregulation of chemokines, cytokines and adhesion molecules and induces endothelial apoptosis (Pober, 2002; Molinar-Inglis et al., 2021). TNF-α signals via two receptors TNFR1 and TNFR2 in endothelial cells, Figure 1A (Slowik et al., 1993). TNF-α signaling is mediated by the adaptor protein TNF-receptor-associated death-domain protein (TRADD) and E3 ligase TNF-receptor-associated factor 2 (TRAF2), which regulate signal transduction pathways associated with endothelial gene transcription, inflammation and apoptosis (Figure 1A). TNF-α activates multiple signaling pathways implicated in endothelial dysfunction including Jun N-terminal kinase (JNK), p38 MAPK and the IκB kinase (IKK) complex that controls activity of activator protein-1 (AP-1), ETS-like-1/3 (ELK1/3), cAMP-response element binding protein (CREB) and nuclear factor kappa B (NF-κB) transcription factors that regulate gene transcription (Figure 1A).

To understand how APC/PAR1 might modulate pathways associated with TNF-α inflammatory signaling, a quantitative phospho-proteomics mass spectroscopy analysis of APC stimulated human umbilical vein endothelial cell (HUVEC)-derived EA. hy926 cells was examined (Lin et al., 2020). Endothelial cells stimulated with APC for 0, 15 or 30 min in three biological replicates were evaluated to determine if APC altered phosphorylation of specific proteins associated with TNF-α signal transduction pathways including known mediators of the NF-κB, AP-1 and p38 MAPK signaling pathways (Figure 1A). NF-κB mediators included NF-κB repressing factor (NKRF), NF-κB activating protein (NKAP), TRAF family member associated NF-κB activator (TANK), and cylindromatosis (CYLD) were detected (Figure 1B). APC stimulation caused a modest decrease in detection of NKAP serine (S)149, S157, and threonine (T)161 phospho-peptides, whereas elevated TANK S225 phospho-peptide and CYLD T393 phospho-peptides were detected basally (Figure 1C). Several AP-1 mediators were detected including JunD, Jun, JunB, activating transcription factor 2 (ATF2), and activating transcription factor 7 interacting protein (ATF7IP) (Figure 1B). In cells treated with APC, we observed a decrease in Jun T239/S243 phospho-peptide and modest increase in ATF7IP S852 and S673 phospho-peptides (Figure 1D). Changes in p38 MAPK mediators following APC treatment were also detected and included MAPK14 (also known as p38 MAPK), CREB binding protein (CBP) and ELK3 (Figure 1B). APC induced increases in MAPK14 T180/Y182 phospho-peptide and CREB BP S1076 phospho-peptide abundance (Figure 1E). Although antibodies to detect most signaling mediator phosphorylation sites do not exist, we were able to examine p38 (MAPK14) phosphorylation induced by APC using anti-phospho p38 T180/Y182 antibodies. APC stimulated p38 phosphorylation (Figure 1F), similar to that observed in the phospho-proteomic analysis. These findings suggest that APC/PAR1 signaling is likely to modulate the function of several TNF-α mediators of the NF-κB, AP-1 and p38 MAPK signaling pathways.

### 3.2 TNF-α induced adhesion molecule expression analysis using endothelial cell RNA-seq datasets

**FIGURE 2:**
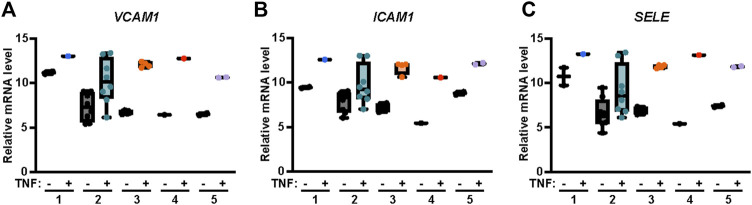
TNF-α induces *VCAM1*, *ICAM1* and *SELE* mRNA expression in endothelial cells. Relative abundance of **(A)** *VCAM1*, **(B)** *ICAM1* and **(C)** *SELE* (E-Selectin) mRNA transcripts in endothelial cells treated with or without TNF-α were obtained from the online repository Endothelial Cell DataBase (EndoDB) endothelial cell RNA-seq transcriptomics datasets. The relative mRNA levels, including replicates, of each of the 5 independent studies were analyzed by box-and-whisker plot with median and range from minimum-to-maximum.

Endothelial cells express several adhesion molecules including VCAM-1, intercellular adhesion molecule-1 (ICAM-1) and E-Selectin, that facilitate leukocyte recruitment and inflammatory responses. Induction of VCAM-1, ICAM-1 and E-Selectin by the cytokine TNF-α is mediated by AP-1, p38 MAPK and NF-κB pathways (Figure 1) (Joyce et al., 2001; Guitton et al., 2011). Thus, we used the publicly available transcriptomics Endothelial Cell DataBase (EndoDB) (Khan et al., 2019) comprised of endothelial cell RNA-seq gene expression datasets to examine TNF-α induced modulation of V*CAM1*, *ICAM1* and *SELE* (E-Selectin) gene expression. We evaluated five independent datasets from studies using human cultured endothelial cells treated with TNF-α including four human umbilical vein endothelial cell (HUVEC) studies (#1, E-GEOD-8166_P2; #2, E-SGRP-3; #3, E-GEOD-2639; and #4, E-GEOD-9055) and one aortic endothelial cell culture study (#5, E-MEXP-3540). TNF-α induced *VCAM1* gene expression in all five independent studies (Figure 2A). A similar result was observed with *ICAM1* and *SELE* gene expression following TNF-α treatment in all five endothelial cell independent studies (Figures 2B, C), supporting the role of TNF-α as a potent inducer of adhesion molecule expression as previously reported (Couffinhal et al., 1993; Burke-Gaffney and Hellewell, 1996; Xia et al., 1998; Viemann et al., 2006).

### 3.3 APC attenuates TNF-α-induced VCAM-1 protein expression but not mRNA stability

**FIGURE 3:**
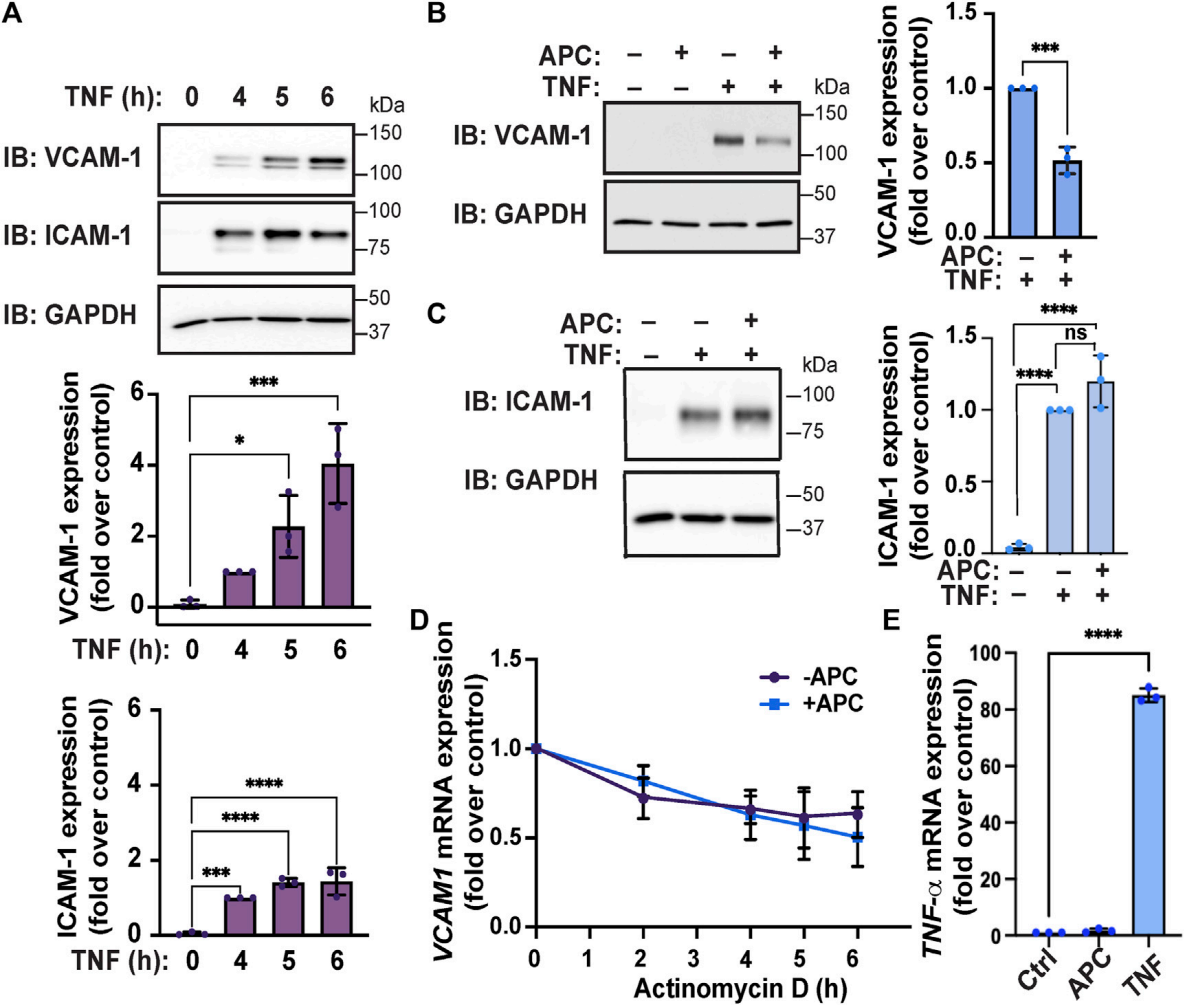
APC attenuates TNF-α-induced VCAM-1 expression in endothelial cells. **(A)** Endothelial cells were stimulated with TNF-α for the indicated times. Cell lysates were immunoblotted for expression of VCAM-1 and ICAM-1 and GAPDH as a loading control. Quantifications of VCAM-1 and ICAM-1 induction are shown as the mean ± S.D. from three independent experiments analyzed by one-way ANOVA; *, *p* < 0.05; ***, *p* < 0.001; ****, *p* < 0.0001. **(B,C)** Endothelial cells were pre-treated with or without APC and then stimulated with or without TNF-α for 24 h. Cell lysates were immunoblotted for VCAM-1, ICAM-1 and GAPDH expression. Quantification of VCAM-1 and ICAM-1 induction (mean ± S.D.) from three independent experiments was analyzed by one-way ANOVA; ***, *p* < 0.001; n. s, not significant. **(D)** Endothelial cells were pre-treated with or without APC, stimulated with TNF-α, and then treated with Actinomycin D for indicated times. Relative VCAM-1 mRNA expression was quantified by RT-qPCR and normalized to 18S ribosomal mRNA expression and is represented as the fold-change relative to TNF-α-induced VCAM-1 expression without Actinomycin D treatment (mean ± S.D.) from three independent experiments. Data were analyzed by one-way ANOVA. **(E)** Endothelial cells were treated with APC or TNF-α for 24 h and changes in TNF-α mRNA transcripts were quantified by RT-qPCR, normalized to 18S mRNA expression and represented as the fold change relative to untreated control. Data (mean ± S.D.) from three independent experiments were analyzed by one-way ANOVA; ****, *p* < 0.0001.

Next, we examined TNF-α-induced VCAM-1 and ICAM-1 adhesion molecule protein expression in endothelial EA. hy926 cells by immunoblot. TNF-α induced a significant increase in VCAM-1 protein expression that remained elevated after 6 h of TNF-α stimulation (Figure 3A). Similarly, a significant increase in ICAM-1 protein expression in endothelial cells was also observed following TNF-α treatment (Figure 3A). To determine how APC/PAR1 modulates TNF-α inflammatory responses, endothelial cells were pretreated with or without APC and then stimulated with TNF-α. In APC pretreated cells, TNF-α-stimulated VCAM-1 protein expression was significantly inhibited (Figure 3B), indicating that APC attenuates cytokine-induced VCAM-1 expression. However, APC pretreatment failed to modulate ICAM-1 expression in HUVEC-derived EA. hy926 cells detected by immunoblot (Figure 3C) and was not further evaluated. We next examined if APC’s capacity to attenuate TNF-α-induced VCAM-1 expression was related to *VCAM1* mRNA stability. In these experiments, endothelial cells were pretreated with APC, stimulated with TNF-α and then incubated with actinomycin D to block gene transcription and the abundance of *VCAM1* mRNA measured over time using RT-qPCR. In the absence of APC pretreatment, an approximately 50% loss of *VCAM1* mRNA was detected after actinomycin D-mediated blockade of gene transcription (Figure 3D). A similar 50% loss of *VCAM1* mRNA was observed in endothelial cells pretreated with APC and actinomycin D (Figure 3D). We further examined the effect of APC pretreatment on TNF-α expression by RT-qPCR and observed a negligible effect (Figure 3E), whereas TNF-α induced a significant increase in TNF-α mRNA transcripts. These data suggest that APC’s capacity to modulate VCAM-1 expression does not occur at the level of VCAM mRNA stability or modulation of TNF-α mRNA expression.

### 3.4 APC/PAR1 requires S1PR1 but not PAR3 coreceptor for protection against TNF-α-induced VCAM-1 expression

**FIGURE 4:**
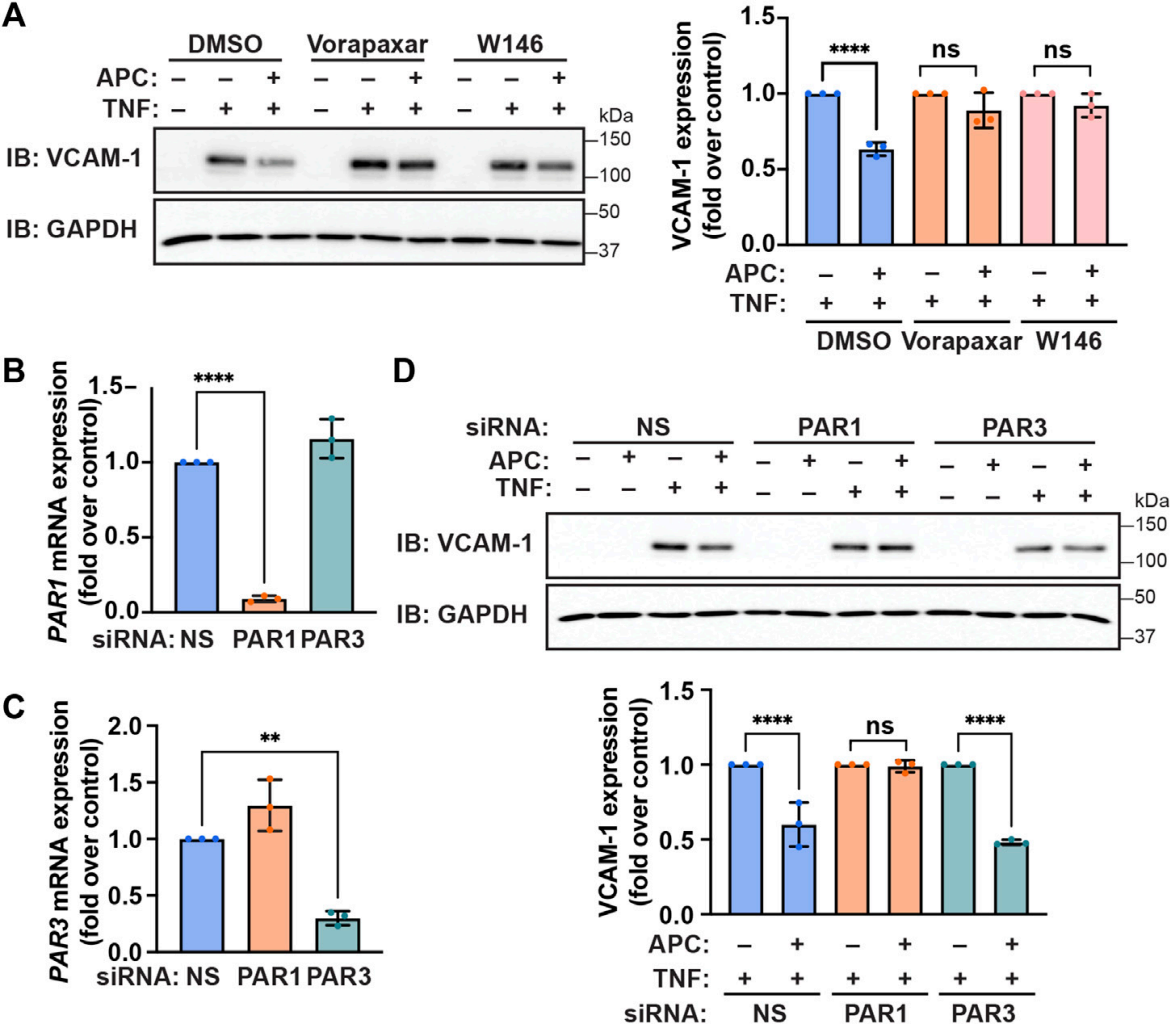
APC-mediated attenuation of TNF-α-induced VCAM-1 expression requires PAR1 and S1PR1. **(A)** Endothelial cells were pre-incubated with Vorapaxar (PAR1 antagonist), W146 (S1PR1 antagonist) or DMSO vehicle control, prior to pretreatment with APC for 30 min and then treated with TNF-α. Cell lysates were immunoblotted for VCAM-1 and GAPDH. Quantification of VCAM-1 induction (mean ± S.D.) from three independent experiments were analyzed by one-way ANOVA; ****, *p* < 0.0001; n. s., not significant. **(B,C)** Endothelial cells were transfected with non-specific (NS), PAR1, or PAR3 specific siRNA. The relative PAR1 **(B)** and PAR3 **(C)** mRNA expression levels were quantified by RT-qPCR. Results were normalized to 18S mRNA expression and are represented as fold-change relative to non-specific siRNA control (mean ± S.D.) from three independent experiments and analyzed one-way ANOVA; **, *p* < 0.01; ****, *p* < 0.0001. **(D)** Endothelial cells were transfected with NS, PAR1, or PAR3 specific siRNA, pretreated with or without APC and then stimulated with TNF-α for 24 h. Cell lysates were immunoblotted for VCAM-1 and GAPDH expression. Quantification of VCAM-1 induction (mean ± S.D.) from three independent experiments was examined by one-way ANOVA; ****, *p* < 0.0001; n. s., not significant.

Previous studies indicate that GPCR co-receptors can distinctly modulate APC/PAR1 mediated cytoprotection (Burnier and Mosnier, 2013; Molinar-Inglis et al., 2021). To validate the role of PAR1 in APC-mediated inhibition of TNF-α-stimulated VCAM-1 adhesion molecule expression, cells were treated with vorapaxar, a human PAR1 selective antagonist. In control cells, APC pretreatment significantly reduced TNF-α-stimulated VCAM-1 protein expression (Figure 4A). However, in cells pre-incubated with vorapaxar, APC failed to attenuate TNF-α induced VCAM-1 protein expression (Figure 4A), suggesting that PAR1 plays a pivotal role in APC-mediated protection against cytokine promoted inflammatory responses. We recently showed that S1PR1 enabled APC/PAR1-mediated protection against cytokine-induced apoptosis (Molinar-Inglis et al., 2021), suggesting that S1PR1 may contribute. Indeed, preincubation with the S1PR1 specific antagonist W146 blocked APC-mediated attenuation of TNF-α-induced VCAM-1 expression (Figure 4A). These data suggest that S1PR1 contributes to APC/PAR1 protection against cytokine induced anti-inflammatory responses.

In addition to PAR1, PAR3 has been shown to function as a co-receptor for APC-mediated barrier protection but its role in anti-inflammatory responses is not known (Burnier and Mosnier, 2013). To determine if PAR3 contributes to APC-mediated protection against TNF-α induced inflammatory responses we used siRNA to deplete cells of PAR3 expression, since there are no effective antagonists. We first optimized the efficiency and specificity of siRNA-targeted PAR1 and PAR3 mRNA degradation in endothelial cells using RT-qPCR. Endothelial cells transfected with the PAR1-specific siRNA caused significant degradation of PAR1 mRNA transcripts but not PAR3 mRNA transcripts measured by RT-qPCR (Figure 4B). Similarly, the PAR3-specific siRNA caused a significant loss of PAR3 mRNA transcripts but not PAR1 mRNA abundance in transfected endothelial cells (Figure 4C). We next examined the contribution of PAR1 and PAR3 to APC-mediated protection against TNF-α-induced VCAM-1 protein expression utilizing the PAR1 and PAR3 specific siRNAs. As expected, APC pretreatment reduced TNF-α-stimulated VCAM-1 protein expression in control non-specific siRNA transfected endothelial cells (Figure 4D). In contrast to control cells, in endothelial cells depleted of PAR1 by siRNA, APC was not able to reduce TNF-α stimulated VCAM-1 expression (Figure 4D). Interestingly, in endothelial cells diminished of PAR3 expression by siRNA, APC retained the capacity to inhibit TNF-α induced VCAM-1 expression (Figure 4D). These data suggest that PAR1 and not PAR3 is the critical mediator of APC-protection against TNF-α-induced VCAM-1 expression.

### 3.5 APC/PAR1 protection against TNF-α induced VCAM-1 expression requires GRK2 and not GRK5

**FIGURE 5:**
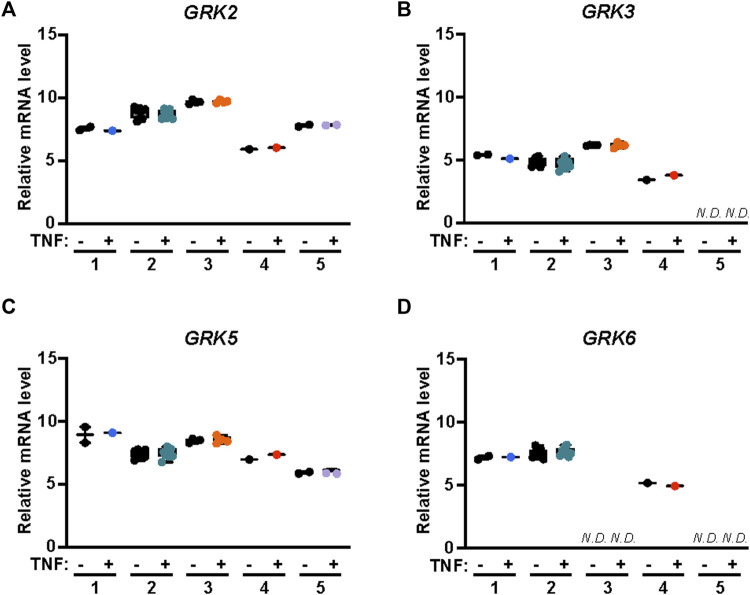
GRK2,3 and GRK5,6 expression in endothelial cells is not modified by TNF-α. GRK mRNA expression was examined in five independent studies including four HUVEC RNA-seq datasets and one aortic endothelial cell RNA-seq data sets treated with or without TNF-α. Data for analysis of GRK2 **(A)**, GRK3 **(B)**, GRK5 **(C)** and GRK6 **(D)** expression in endothelial cells with or without TNF-α treatment were obtained from Endothelial Cell DataBase (EndoDB). The relative mRNA levels, including replicates, of each of the 5 independent studies were analyzed by box-and-whisker plot with median and range from minimum-to-maximum. N. D is not detected.

APC/PAR1 cellular signaling is mediated primarily by the β-arrestin-2 isoform, which drives most if not all aspects of endothelial cytoprotection (Soh and Trejo, 2011; Roy et al., 2016; Kanki et al., 2019; Molinar-Inglis et al., 2021). GRKs are known to mediate phosphorylation of activated GPCRs and facilitate the recruitment of β-arrestins. To determine which GRKs function in APC/PAR1 protection against TNF-α mediated inflammatory responses, we first used the publicly available transcriptomics Endothelial Cell DataBase (EndoDB) RNA-seq gene expression datasets to examine GRK expression and potential modulation by TNF-α in five separate RNA-seq studies including four HUVEC datasets and an aortic endothelial cell dataset. GRK5 and GKR2 appeared to be more highly expressed in human endothelial cells compared to GRK3 and GRK6 expression (Figures 5A–D), similar to that previously reported for GRK expression in cardiac tissue (Rockman et al., 1996; Penela et al., 2003; Dzimiri et al., 2004; Matkovich et al., 2006). Interestingly, TNF-α failed to modulate expression of the highly expressed GRK5 and GRK2 as well as the moderately expressed GRK3 and GRK6 in human endothelial cells (Figure 5), suggesting that GRK gene expression is not regulated by the pro-inflammatory TNF-α cytokine.

**FIGURE 6:**
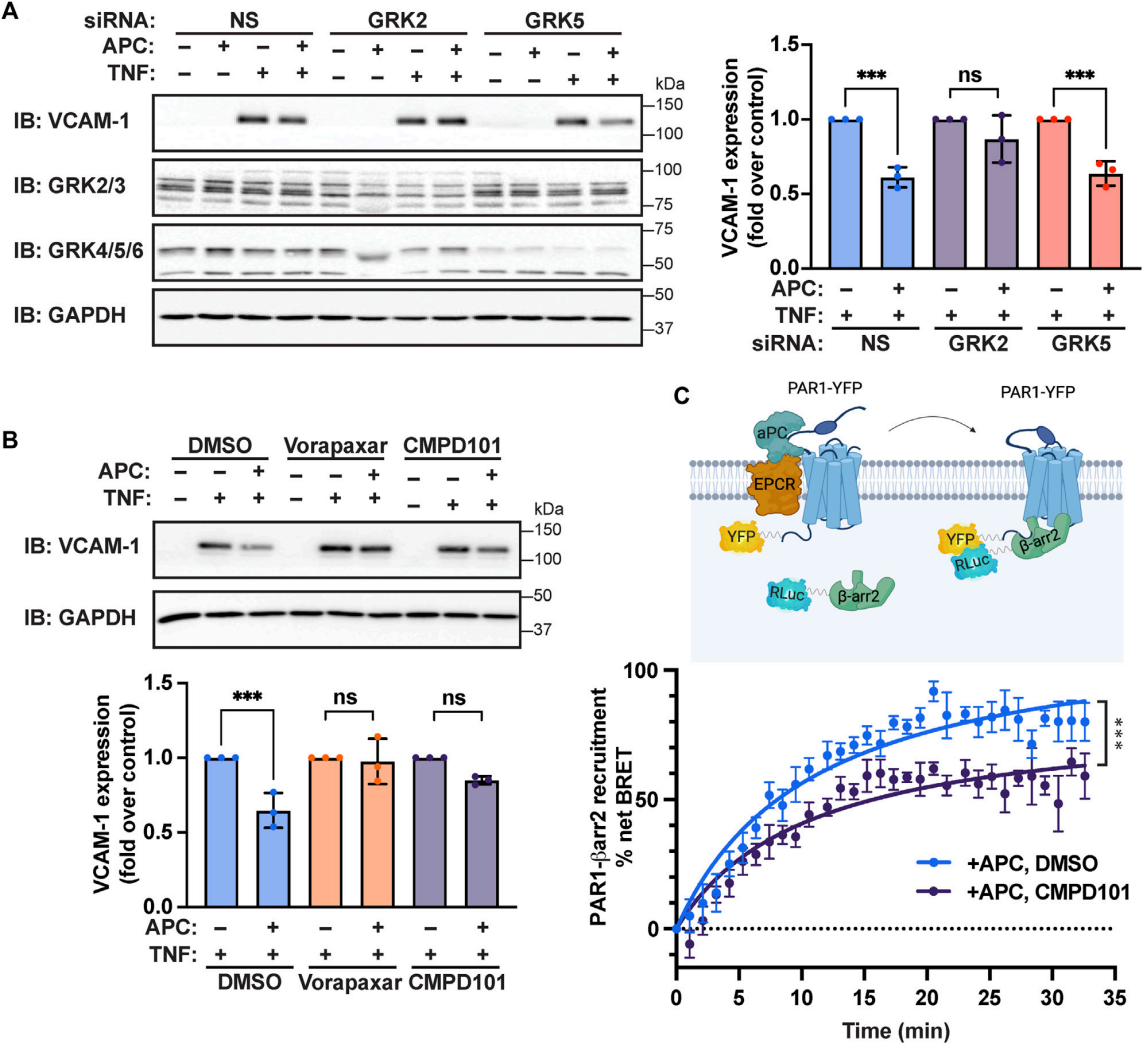
APC-mediated attenuation of TNF-α-induced VCAM-1 expression requires GRK2. **(A)** Endothelial cells transfected with non-specific (NS), GRK2, or GRK5 siRNA were pretreated with or without APC and then stimulated with TNF-α. Cell lysates were immunoblotted for VCAM-1, GRK2/3, GRK4/5/6 and GAPDH expression. Quantification of VCAM-1 expression (mean ± S.D.) from three independent experiments were analyzed by one-way ANOVA; ***, *p* < 0.001; n. s., not significant. **(B)** Endothelial cells preincubated with Vorapaxar (PAR1 antagonist) or CMPD101 (GRK2 inhibitor) were pretreated with APC and then stimulated with TNF-α. Cell lysates were immunoblotted for VCAM-1 and GAPDH. Quantification of VCAM-1 expression (mean ± S.D.) from three independent experiments were analyzed by one-way ANOVA; ***, *p* < 0.001; n. s., not significant. **(C)** HEK293 cells transfected with PAR1-YFP and β-arr2-Rluc coexpressing EPCR were pretreated with CMPD101 or DMSO vehicle control, stimulated with APC and β-arr2 recruitment to PAR1 was determined by BRET. Data (mean ± S.D.) from three independent experiments were analyzed by one-way ANOVA; ***, *p* < 0.001.

**FIGURE 7:**
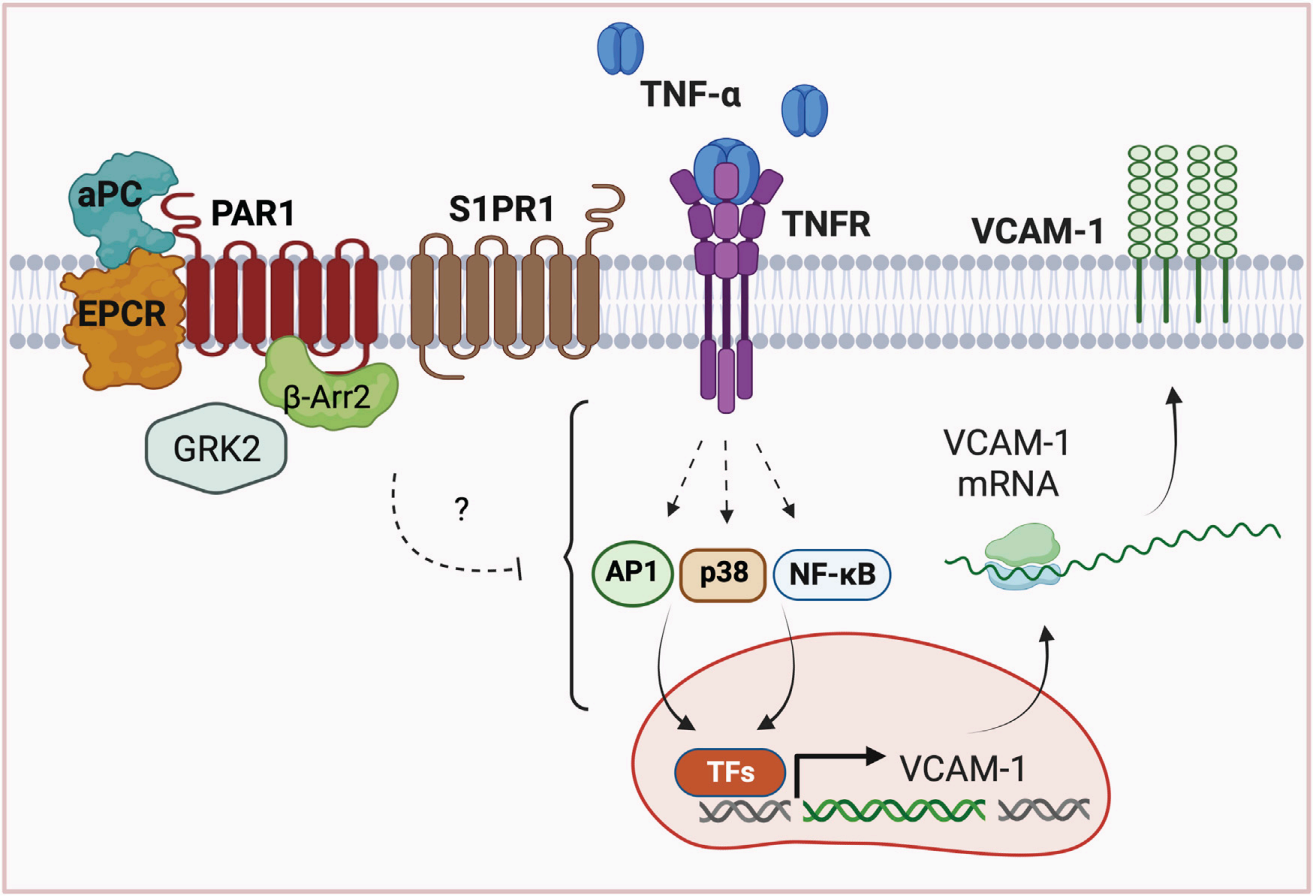
Model of APC/PAR1-S1PR1 mediated protection against TNF-α induced VCAM1 expression. In endothelial cells, APC bound to EPCR cleaves and activates PAR1 to stimulate β-arr2 mediated cytoprotection against TNF-α induced upregulation of adhesion molecule VCAM-1 expression. Interestingly, APC/PAR1-mediated cytoprotection against TNF-α-induced VCAM-1 expression further requires the co-receptor S1PR1 and unexpectedly is dependent on GRK2. These studies highlight the complexity of APC/PAR1 cytoprotective signaling, which utilizes multiple co-receptors and different GRKs to promote distinct β-arr2-dependent cytoprotective responses in endothelial cells. The mechanism by which APC/PAR1 signaling intersects with the TNF-α-stimulated AP-1, p38 MAPK and NF-κB pathways to promote gene transcription of VCAM-1 expression is not known.

To assess the role of GRKs in APC/PAR1 protection against TNF-α-stimulated inflammatory responses, we first used siRNA to deplete endothelial cells of endogenous GRK5 and GRK2 expression. Surprisingly, APC-mediated attenuation of TNF-α-stimulated VCAM-1 expression was virtually ablated in endothelial cells deficient in GRK2 expression, compared to control non-specific siRNA transfected cells (Figure 6A). In addition, depletion of endogenous GRK5 expression failed to effect APC’s capacity to attenuate TNF-α stimulated VCAM-1 expression (Figure 6A), consistent with a preferential role for GRK2. To further examine the role of GRK2 in APC/PAR1 endothelial anti-inflammatory responses, we utilized CMPD101, a membrane permeable selective inhibitor of GRK2/GRK3 activity. In DMSO vehicle pretreated endothelial cells, APC exhibited robust cytoprotective against TNF-α stimulated VCAM-1 expression (Figure 6B), which was blocked by the PAR1 antagonist vorapaxar. However, in CMPD101 pretreated endothelial cells, APC failed to protect against TNF-α-induced VCAM-1 expression (Figure 6B). To further evaluate GRK2 function, the role of GRK2 in APC-induced β-arr2 recruitment to PAR1 was examined using BRET assays in HEK293 cells. In control DMSO pretreated cells, APC stimulated an increase in β-arr2 recruitment to PAR1 that appeared to peak at 20 min (Figure 6C). In contrast, APC-stimulated β-arr2 recruitment to PAR1 was significantly inhibited in CMPD101 treated cells (Figure 6C). Together these studies report an unexpected regulation of APC/PAR1-mediated protection against TNF-α-induced VCAM-1 expression that is mediated by PAR1/S1PR1 and GRK2 and likely controlled by AP-1, p38 MAPK and NF-κB transcription factor pathways (Figure 7).

## 4 Discussion

In this study, we define a new pathway by which APC modulates TNF-α cytokine induced VCAM-1 expression in human cultured endothelial cells. We found that APC stimulation alters phosphorylation of several TNF-α signaling mediators associated with AP-1, p38 MAPK and NF-κB transcription factor pathways assessed by mass spectrometry. In addition, we verified using a repository of endothelial cell RNA-seq datasets that TNF-α induces expression of VCAM-1, ICAM-1 and E-Selectin, which are known to be transcriptionally regulated by AP-1, p38 MAPK and NF-κB pathways and mediate leukocyte recruitment and inflammatory responses (Pober, 2002). We further show that APC/PAR1 attenuates TNF-α-induced VCAM-1 expression independent of mRNA stability. Using a combination of pharmacological inhibitors and siRNAs, we demonstrate a critical role for S1PR1 and GRK2 in APC/PAR1 protection against TNF-α induced VCAM-1 expression. This study suggests that different APC/PAR1 cytoprotective responses are mediated by discrete β-arrestin-2-driven signaling pathways that are specified by distinct co-receptors and GRKs.

An important finding from our study is that different GPCR co-receptors drive distinct β-arrestin-2 mediated cytoprotective responses. Co-receptor interaction is known to alter GPCR-induced β-arrestin biased signaling (Shen et al., 2018), but is understudied in biased signaling. We found that GPCR co-receptor interaction is relevant to APC/PAR1 cytoprotection. Here we show that S1PR1 functions as a GPCR co-receptor to facilitate APC/PAR1’s ability to suppress TNF-α induced VCAM-1 expression (Figure 4), an anti-inflammatory response. These findings are consistent with S1PR1 capacity to decrease leukocyte adhesion molecule expression (Galvani et al., 2015; Burg et al., 2018). Previous studies established a role for PAR3 in APC/PAR1 mediated endothelial barrier protection *in vitro* (Burnier and Mosnier, 2013). Other studies demonstrated a function for PAR3 in APC/PAR1-mediated neuroprotection and cytoprotective signaling in podocytes *in vivo* (Guo et al., 2004; Guo et al., 2009; Madhusudhan et al., 2012). While S1PR1 has been shown to contribute to basal integrity of the endothelial barrier (Feistritzer and Riewald, 2005), the role of S1PR1 in APC/PAR1-induced cytoprotective responses are less clear. We recently showed that S1PR1 is required for APC/PAR1-induced Akt-mediated anti-apoptotic activities induced by TNF-α in human cultured endothelial cells (Molinar-Inglis et al., 2021). Here, we now show that S1PR1 is also necessary for APC/PAR1 protection against TNF-α-stimulated VCAM-1 expression, suggesting common regulatory pathways. These studies indicate that different GPCR co-receptors modulate distinct APC/PAR1 cytoprotective responses.

The mechanisms by which GPCR co-receptors influence APC/PAR1-stimulated β-arrestin-2-mediated downstream signaling are not well defined. In previous work, we reported that APC/PAR1 signals independent of Gi protein (Soh and Trejo, 2011) but rather activates β-arrestin-2-dependent polymerization of dishevelled-2 (Dvl-2), ERK1/2 and Rac-1 activation and endothelial barrier protection (Soh and Trejo, 2011). These studies suggested that β-arrestin-2 and Dvl-2 might function as universal scaffolds to mediate most, if not all APC/PAR1 cytoprotective responses, but this is not the case. In recent work, we found that β-arrestin-2 and not Dvl-2 is essential for APC-stimulated sphingosine kinase-1 activation of S1PR1 and Akt signaling, which protects against TNF-α induced cell death (Molinar-Inglis et al., 2021). In addition, APC/PAR1-activated S1PR1 signaling did not intersect with APC-stimulated β-arrestin-2-mediated ERK1/2 signaling (Molinar-Inglis et al., 2021), consistent with discrete β-arrestin-2 regulated signal transduction pathways. Interestingly, a previous study showed APC-induced β-arrestin-2- and Dvl-2-mediated inhibition of cytokine-induced endothelial-monocyte adhesion, an anti-inflammatory response (Roy et al., 2016). However, further investigation is needed to determine how Dvl-2 contributes to APC suppression of cytokine induced monocyte recruitment.

We found that APC modulates multiple TNF-α regulated signaling components that control AP-1, p38 MAPK and NF-κB transcription factor pathways based on our mass spectrometry analysis (Figure 1), which is consistent with reported studies (Joyce et al., 2001; Guitton et al., 2011). However, it is not known how APC/PAR1 signaling modulates AP-1, p38 MAPK and NF-κB regulated transcription factor pathways to control VCAM-1 expression induced by TNF-α, while not affecting TNF-α-stimulated ICAM-1 expression. The VCAM-1 and ICAM-1 gene promoter regions contain several inducible transcription factor binding sites including sites for AP-1, NF-κB, Sp1 and other transcription factors (Cook-Mills et al., 2011). In addition, TNF-α signals through multiple pathways including AP-1, p38 MAPK and NF-κB to regulate VCAM-1 and ICAM-1 gene expression (Figure 1), but precisely how these pathways converge to enable APC to suppress cytokine-induced VCAM-1 and not ICAM-1 expression in HUVEC-derived EA. hy926 cells is not known (Figure 7). Our findings indicate that APC/PAR1 likely controls VCAM-1 gene transcription since APC failed to modulate VCAM-1 mRNA stability (Figure 3D), consistent with APC/PAR1 phosphoproteome association with processes such as DNA binding and chromatin regulators identified in gene ontology analysis (Lin et al., 2020).

Classic biased agonists use different GRKs to distinctly phosphorylate activated GPCRs, which promotes different β-arrestin binding modes and functions (i.e. desensitization, internalization and signaling) (Kim et al., 2005; Ren et al., 2005; Shenoy et al., 2006; Drube et al., 2022). Of the seven GRKs, GRK2/GRK3 and GRK5/GRK6 are ubiquitously expressed and relevant to endothelial biology (Tiruppathi et al., 2000; Roy et al., 2016). Indeed, analysis of endothelial RNA-seq datasets indicate that GRK5 and GKR2 are highly expressed in human endothelial cells (Figure 5). GRK5 was shown previously to desensitize thrombin/PAR1 signaling in human cultured endothelial cells (Tiruppathi et al., 2000). However, the function of GRKs in regulation of APC/PAR1 cytoprotective responses is not well understood. A previous study implicated GRK5 in APC/PAR1 protection against endothelial barrier permeability (Roy et al., 2016). Intriguingly, we found that GRK2 and not GRK5 mediates APC/PAR1 protection against TNF-α induced VCAM-1 expression (Figure 6). However, the regulation of APC/PAR1 cytoprotective signaling is more complex with GRK5 displaying dual functions controlling both thrombin signaling as well as APC/PAR1 cytoprotective signaling. The mechanism responsible for divergent GRK activity at APC-activated PAR1 is not known. In addition, our studies suggest that both GRK5 and GRK2 can promote APC/PAR1-stimulated β-arrestin-2-dependent signaling. However, it is unclear if GRK5 and GRK2 have additional roles is desensitization of either APC-activated PAR1 or the S1PR1 receptor.

## 5 Conclusion

In summary, this study illustrates the complexity of APC/PAR1 β-arrestin-2 signaling that is controlled by different GPCR co-receptors and GRKs that drive distinct endothelial cytoprotective responses. Understanding APC/PAR1 signaling pathways that enable the endothelium to acquire resilience to resist injury and disruption is critical for the advancement of new targets for therapeutic development.

## Data Availability

The datasets presented in this study can be found in online repositories. The names of the repository/repositories and accession numbers can be found below: http://www.proteomexchange.org/, PXD016368; https://massive.ucsd.edu/ProteoSAFe/, MSV000084604.
